# Raising to the Challenge: Building a Federated Biobank to Accelerate Translational Research—The University Biobank Limburg

**DOI:** 10.3389/fmed.2019.00224

**Published:** 2019-10-22

**Authors:** Loes Linsen, Kimberly Vanhees, Evi Vanoppen, Kim Ulenaers, Suzanne Driessens, Joris Penders, Veerle Somers, Piet Stinissen, Jean-Luc Rummens

**Affiliations:** ^1^University Biobank Limburg (UBiLim), Hasselt, Belgium; ^2^Faculty of Medicine and Life Sciences, Limburg Clinical Research Center, Hasselt University, Diepenbeek, Belgium; ^3^Clinical Laboratory, Jessa Hospital, Hasselt, Belgium; ^4^Faculty of Medicine and Life Sciences, Biomedical Research Institute, Hasselt University, Diepenbeek, Belgium; ^5^Clinical Laboratory, Hospital East-Limburg (ZOL), Genk, Belgium

**Keywords:** UBiLim, quality, biobank, multi-disciplinary, translational research

## Abstract

Irreproducibility of research results is one of the major contributing factors to the failure of translating basic research results into tangible bedside progress. To address this, the University Biobank Limburg (UBiLim) was founded by a collaboration between Hasselt University, the Hospital East-Limburg, and the Jessa Hospital. This paper describes the evolution of this process and the barriers encountered on the way. UBiLim evolved from an archival collection over a single-site biobank into a federated structure, supporting translational research at the founding institutions. Currently, UBiLim is a federated biobank, with an established organizational structure and processing, and storage facilities at each of the three sites. All activities are integrated in an ISO15189-accredited Quality Management System and based on (inter)national biobank guidelines. Common methods for processing and storage of a plethora of sample types, suitable for state-of-the-art applications, were validated and implemented. Because the biobank is embedded in two hospitals, the request of researchers to include certain sample types or enroll specific patient groups can quickly be met. Funding has been a major challenge in each step of its evolution and remains the biggest issue for long-term biobank sustainability. To a lesser extent, the Belgian legislation and the operational cost of information management system are also concerns for smooth biobank operations. Nonetheless, UBiLim serves as a facilitator and accelerator for translational research in the Limburg area of Belgium that, given the fields of research, may have an impact on international patient care.

## Introduction

Despite major advances in life sciences and medical technologies, there often is a large gap between basic science outcomes and their translation into the clinic (1). A major contributing factor is the irreproducibility of preclinical research results, with one of the primary causes being the quality of the biological reagents and reference materials used (2). Consequently, many researchers now question the validity of their previous findings because of concerns about the quality of the biospecimens (3). In the last decades, these observations led to the development of modern biobanking, with a focus on improved biospecimen quality through a more professional operational development (4). Furthermore, biobank networks were established to facilitate the acquisition of a higher number of biospecimens in a shorter amount of time to meet the increasing scale and complexity of research studies (5). Scandinavia has a headstart in establishing these networks because of their long tradition of large-scale biobanking combined with comprehensive, population-based health data registries linkable to unique personal identifiers, enabling follow-up studies spanning many decades (6). In 2004, these Nordic biobanks partnered together in the “Cancer control using population-based registries and biobanks (CCPRB)” project. The goal of this project was to facilitate and improve cancer research by combining biobank samples and registry data and to establish Good Biobanking Practices (7). The partner “Limburg Cancer Registry (LIKAR)” was incorporated into the network because of its pioneering and state-of-the-art cancer registration practices in the Belgian province of Limburg (8, 9). For the hematology data of the registry, it relied on a close collaboration with the Virga Jessa Hospital (Hasselt, Belgium), which routinely stored bone marrow (BM) smears used for the registration. In 2006, the CCPRB consortium decided to transform the ongoing hematology collection into an actual biobank. This was the founding step for the creation of the translational research supporting University Biobank Limburg (UBiLim), a collaboration between two regional hospitals and a university. This paper describes the evolution of an archival collection into a professional, federated biobank structure that successfully supports multi-domain translational research through provision of qualitative sample processing, storage, and distribution activities. It also highlights the barriers that were overcome at each stage.

## Results and Discussion

### From Archive to Local Biobank: Starting Small but Aiming High

The systematic storage of stained BM smears in the clinical laboratory of the Virga Jesse Hospital had started in the mid-nineties. The archive was built to contain samples from all BM punctures routinely performed at or sent to the hospital. The scientific potential of this archive for hematological diseases was anticipated, also because of the presence of precursor stages of malignant diseases and the possibility to capture potential aggressive transformation events (10). Within the context of the CCPRB project, this archival collection was transformed into a biobank [defined here as a structured facility that receives (processes), stores, and distributes biospecimens coupled to associated (clinical) data and with all aspects (including personnel, infrastructure, etc.) managed according to professional and quality standards]. To this end, the collection was expanded from 2007 onwards to contain additional sample types such as plasma and white blood cell pellets from peripheral blood and BM, stored at −80°C. Additionally, the biobank processes were developed in accordance to the ISO 15189 accreditation of the laboratory to already ensure a high standard of all aspects of work (also including document/record management, training, etc.). Furthermore, sample processing methods were validated prior to use to provide fit-for-purpose samples (11). Finally, an in-house built sample management system was used to capture a limited set of donor and sample data on the samples. This multifaceted approach provided a clear added value to the quality and integrity of the sample, allowing for future research with more demanding needs.

The biggest challenge in the transformation to a local biobank was the availability of the necessary financial resources. All of the above was initially in the hands of one operational biobank manager, supported by the clinical lab director, and eventually supported by trained lab technicians for the actual sample processing. Generally, only 37% of costs were funded by the CCPRB project, while 63% of the cost was taken by the hospital. Personnel cost had the highest impact, with a 2:1 ratio to other costs (consumables, equipment, and miscellaneous). In this initial phase, the average operational cost was about €100.000, about half of what has been reported for biobanks of similar (budget) size and time in operation (12). This difference can probably be explained by the embedded nature of the biobank in the clinical laboratory, allowing the use of common facilities.

The sample management system built in-house proved to be an additional challenge. Initially, it had been set up by the local IT department to accommodate the processing and storage phase of the sample. However, it quickly became obvious that coverage of the pre-collection and post-storage phase (sample distribution, assign studies and projects, informed consent management, etc.) was also required to support the complete set of biobank activities. This required additional configuration, which was not budgeted for, and increased the dependency on the IT department imposing a higher risk of failure (13, 14). Given the selection of a commercial system within the CCPRB project, the in-house system was not developed further in attendance of implementation of the commercial system.

**Figure 1:**
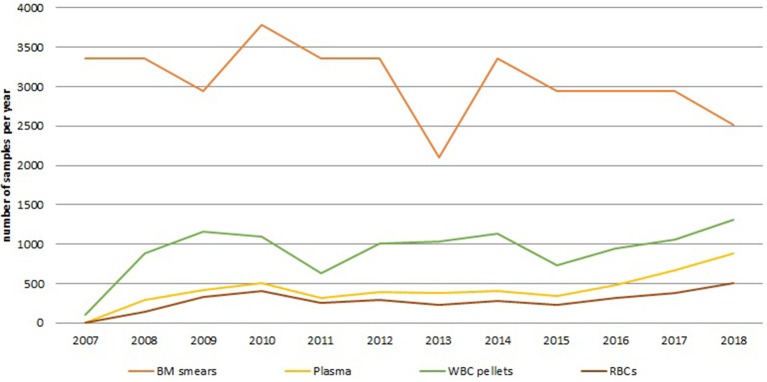
Annual number of samples for the different sample types collected within the hematology collection since the start of the biobank to December 2018. The orange line shows the number of BM smear samples, the yellow line shows the number of plasma samples, the green line shows the number of white blood cell pellets, the brown line shows the number of red blood cell samples. BM, bone marrow; RBCs, red blood cells; WBC, white blood cell.

By 2009, at the end of the CCPRB project, a local biobank focusing on a single disease domain had successfully been established from an operational point of view. In addition to 39,060 bone marrow smears, 3,313 samples were available for research, representing twenty different hematological diagnoses. Figure 1 shows the number of samples gathered in this collection yearly until December 2018. Initially, a sharp increase in sample number of the newly added sample types can be observed, as can be expected from process optimization to improve collection rates. A fairly stable number of samples were collected afterwards, with a trend of increased numbers showing for the last 4 years. This results from expanding the collection to incorporate paired blood samples and by introducing a smaller container type in 2017. Currently, the collection holds 60,897 samples in total and their fitness-for-purpose for omics-technologies has been demonstrated (15).

### From Local Biobank to Federated Biobank: The Governmental Phase

Mid-2008, different biobank funding schemes were launched in Belgium: the National Cancer Plan and the Center for Medical Innovation organized by the federal and regional government, respectively. To increase the chances of obtaining financing for the biobank activities, a collaboration was set up between the two biggest regional hospitals of Limburg: the Virga Jesse Hospital, by then renamed to the Jessa Hospital, and the Hospital of East-Limburg, together with Hasselt University to form the University Biobank Limburg (UBiLim). Given the different physical locations, it was decided to construct a federated biobank with processing and storage facilities at the three sites. Its structure and processes were centrally controlled but allowed federated sample management according to harmonized procedures. However, despite the prominent and qualitative nature of the biobank, no direct funding for UBiLim could be obtained from the National Cancer Plan. A two-phase funding was granted for 4 years (2009–2013) by the local government to strengthen the collaboration by setting up a common framework and evolve to a federated biobank. The setup was built from the biobank platform already present at the Jessa Hospital, integrating the “external” activities into one structure according to the requirements of the existing biobank quality management system (i.e., training, document management, etc.). The activities were expanded further to include method validation for new sample processing procedures (16). Additionally, the performance of these procedures was assessed by participation in biobank-specific proficiency testing schemes since 2011 (17).

**Figure 2:**
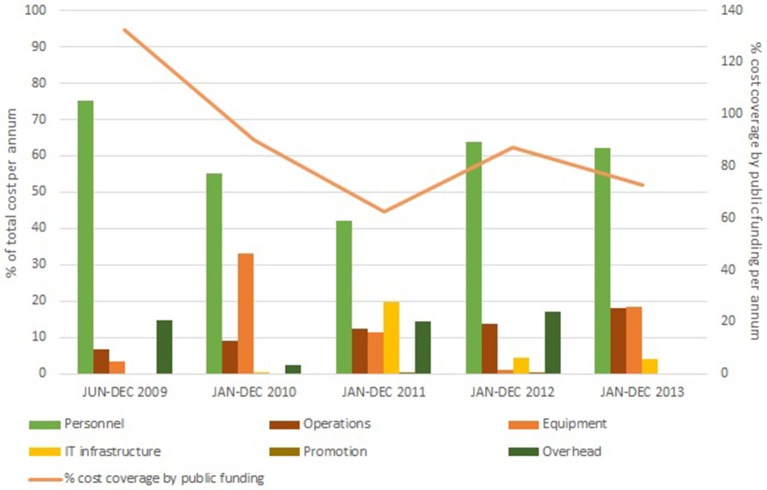
Annual cost distribution in the federated biobank phase (June 2009 to December 2013). Costs are subdivided in the six main accountancy cost categories: Personnel (green bar), IT infrastructure (yellow bar), Operations (brown bar), Promotion (olive bar), Equipment (orange bar), and Overhead (dark green bar). Percentage of cost coverage by public funding is displayed on the secondary *Y*-axis (orange line).

The available funding covered 80% of total costs of UBiLim (Figure 2). Fifty-six percent of the available budget was used to maintain the biobank personnel at the Jessa hospital and to gradually expand it with dedicated biobank technicians at the newly added sites of Hasselt University and the Hospital East-Limburg. Twenty-two percent of funding was invested in additional storage capacity, equipment for sample processing and quality control, and the local acquisition of a commercial biobank information management system accessible from the three sites. Operational costs averaged at 12% of total costs. Annual total costs tripled compared to the earlier phase of the biobank, due to increase of activities and associated needs for personnel and equipment. These numbers are comparable to those recently reported for international biobanks of similar size and time in operation (12). This study also indicated that on average up to 20% of biobank costs is covered by institutional funding. It should be noted, however, that the majority of biobanks in this study was US based, where dependency on publicly funded clinical research is higher compared to the other international respondents. In Belgium, however, the biobanking activities have not been incorporated in the national health program, generating a situation similar to the US. Nevertheless, the potential cost and availability of funding source aspects should be taken into consideration when starting up a biobank and demonstrate the need for support by the institution housing to achieve operational biobank success.

During this 4-years period, UBiLim's in-house built sample management system was replaced by the biobank information management system Labvantage–Sapphire, configured to meet the common biobank needs within the CCPRB project. Although this decision came with a large upfront cost, several arguments supported this conclusion: the completeness of the system regarding biobank processes, the ready-to-deploy state, the modularity to maintain flexibility, the stability of the system to contain several thousands of data, and the web application allowing external access. Typically, the first two items are decisive to choose for commercial solutions (13). The system's installation and additional customization costs attributed to 7.5% of total costs of biobank operations during this 4-years period (Figure 2). Actual implementation and validation at the biobank required additional configuration by a trained biobank super-user. This super-user commitment takes on average about 10% of personnel time for the overall period, similar to findings reported elsewhere (12). While training a dedicated biobank employee to super-user level requires additional investment, it reduces long-term running costs by avoiding expensive programmer hours for small adaptations. Furthermore, the inter-institutional access to this system, while allowing enough flexibility to cover site-specific needs, accelerated the harmonized approach ensuring the same quality standard across the three sites.

Consistent with best practices in the field, a governance structure was established at UBiLim, consisting of a steering committee, a scientific review board, and a management group, membered by representatives of the three institutes (18–20). The challenge in the composition of the scientific review board was to represent all relevant disciplines, while maintaining a manageable group size and institutional representation. This was achieved by a preselection of members by the management group, proposed to and approved by the institutional directors, which allowed immediate buy-in from all researchers and is a reported shared success factor for biobanks globally (21, 22). An access policy was set up, allowing access to internal and external researchers of both academic and commercial affiliation upon approval of (ethical review board approved) projects by the scientific review board based on the evaluation of scientific validity, sample prioritization, and funding. To protect the interest of the original sample collector, up to 5 years of exclusive access can be granted. Custody of the samples however remains at UBiLim. This approach is in line with the ethical and scientific consensus regarding access policies for biobanks improving/facilitating sample and data sharing for global health and again accentuates the progressive role UBiLim played in the Belgian landscape (23–28). In parallel, a national law was released in 2008 stating the conditions regarding the collection and use of human bodily material for therapeutic or research use (29). While primary and secondary use of samples for further research requires consent from the donor, an exception is made for leftover samples from clinical practices where an opt-out system is put in place. Although the biobank aspect of this law did not come into effect until 2018, the necessary processes were already put in place to accommodate these requirements.

### From Federated Biobank to Translational Research Supporting Facility: Bridging the Gap

The next step in the evolution of UBiLim was to use its federated biobank approach to facilitate translational research. To accelerate the translation of innovations in health care into practical applications, the Flemish government invested in the establishment of the Flemish Biobank in 2009, by the foundation of the Center of Medical Innovation (CMI, now the Flemish Biobank Network). The primary approach was to set up professional biobank facilities in four Clinical Research Centers and centralizing the data in a virtual Flemish biobank catalog to allow increased visibility, use, and sharing of biospecimens. Five focus domains were identified, with Hasselt University heading the activities of the Rheumatology focus group (the others being Sudden death, Hepatotropic viruses, Diabetes, and Inflammatory bowel disease) and UBiLim acting as Hasselt University's central biobank. Each CMI affiliate collected samples according to agreed harmonized quality guidelines and procedures within the CMI network, while samples remained under control of the collecting institution. This approach was similar to the Dutch String of Pearls Initiative, which has proven to be a successful method in addressing translational research challenges (30, 31). With Hasselt University/UBiLim not acknowledged as a full-blown center within the CMI project, only partial funding could be obtained for operational activities and participation in the development of the centralized catalog. Nevertheless, UBiLim passed a peer-review audit, set up within the CMI to verify the quality status of the affiliated biobanks, with flying colors despite being the only complex federated biobank in the project. Unfortunately, even though all key deliverables of the project were realized, funding for the CMI was discontinued in 2015 due to the changing political landscape, resulting in the abolition of the project. However, the rheumatology focus group for instance continues to collaborate through sample sharing to this day at their own expense, albeit at a slower pace, highlighting the strength of the project, and the resilience of the affiliated members (32).

**Figure 3:**
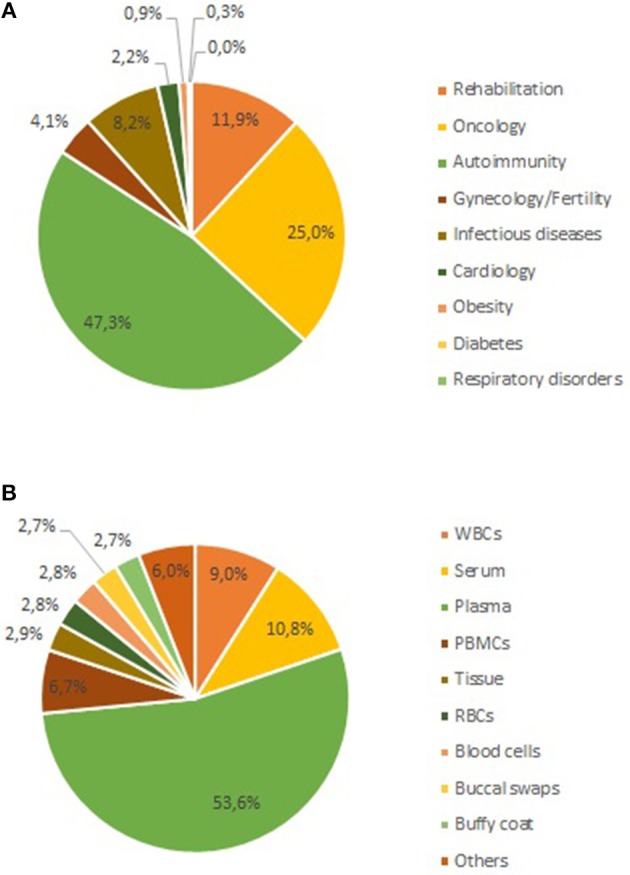
Current sample content of the UBiLim catalog in December 2018. **(A)** Shows the proportional distribution of stored samples per research domain, and **(B)** shows the distribution of stored samples per sample type. PBMCs, peripheral blood mononuclear cells; RBCs, red blood cells; WBC, white blood cell.

In parallel to the CMI project, the three institutes that conceived UBiLim also set up the Limburg Clinical Research Program (LCRP) in 2010. Funding for this project was obtained from the Flemish and local provincial governments to enhance local innovation, health care, and education. Five research domains were defined in the LCRP program as key focus areas for project-based research (cardiology, oncology, anesthesia/neurology, gynecology/fertility, and infection diseases/immunity). Several of the research projects within the LCRP domains were in need of biobank support for their activities. Given its unique position, UBiLim could act as a facilitator for these projects. Furthermore, with the expertise gained, it also contributed to the further improvement of study quality from a biospecimen perspective. From 2014 onwards, part of the LCRP funding was hence invested in the UBiLim operations to accommodate for the heightened activities and secure the number of staff as set out for the federated approach. Not all LCRP projects require biospecimens for their research; however, when human bodily material is collected within the LCRP projects, it is processed and/or stored in the UBiLim facility. As a result, some key collections for translational research are present in the biobank (33–36). Figure 3A shows the current representation of samples for all research domains currently present in the inventory (including LCRP and CMI domains). About half of the samples are related to autoimmune diseases (48%), followed by oncology (25%), and rehabilitation sciences (12%). This is not unexpected given the long-standing focus on auto-immune diseases of Hasselt University and its CMI-related incorporation in UBiLim. The oncology collection mainly consists of the hematology collection forming the foundation of UBiLim. Different types of samples are being collected, with plasma samples making up 53% of collected samples (51% heparin plasma, 20% EDTA plasma, and 29% either citrate or oxalate plasma), serum 11%, and white blood cell and PBMC fractions 9 and 6%, respectively (Figure 3B). Tissue samples represent only 3% of the biospecimens present, resulting from the biobank originally embedded in a clinical laboratory, not a pathology department. Finally, UBiLim also stores some rare sample types, such as skin tapes to strip the stratum corneum of low-level laser-treated cancer patients to investigate inflammation pathways.

**Figure 4:**
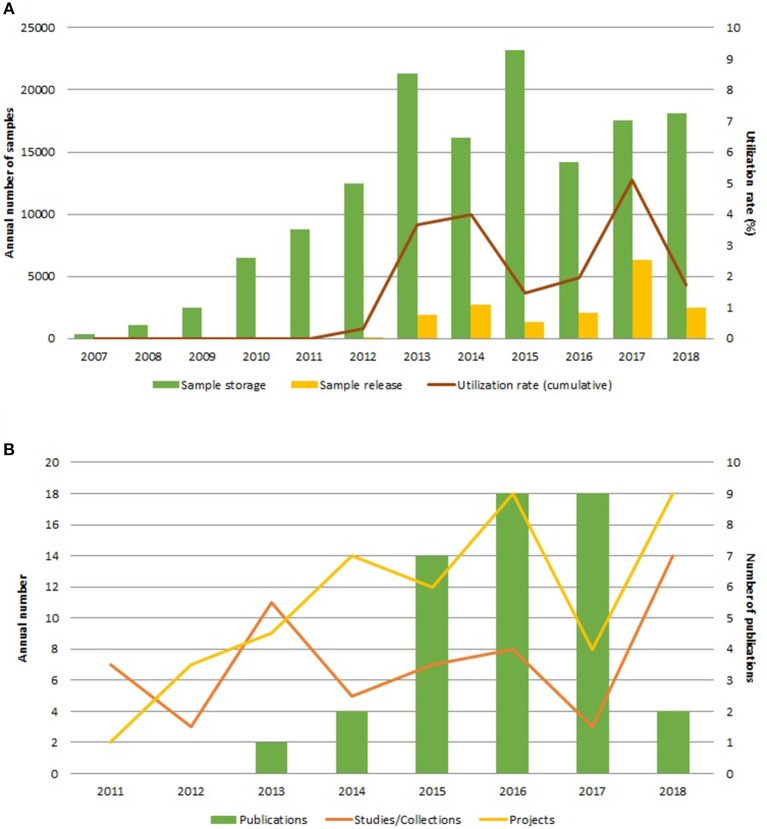
Overview of the sample-based activities, cumulated for all sample collections of UBiLim. **(A)** Annual number of samples stored (green bar) and released (yellow bar) and annual utilization rate of samples (brown line, displayed on secondary *Y*-axis). **(B)** Annual number of studies/collections setup (orange line) and projects for sample use started (yellow line) by UBiLim and annual number of publications using samples derived from UBiLim (green bars, displayed on the secondary *Y*-axis).

Already by the end of 2013, UBiLim was effectively supporting the translational research community's biobank needs, as evidenced in Figure 4A. Since 2007, on average 11,900 samples are stored and about 1,400 samples were distributed annually. The number of studies (collections starting) and projects (use of samples) shows on average an upward trend (Figure 4B). The observed average utilization rate of 1.5% is lower than that reported for classic biobanks (37). Since most collections are still in their “exclusivity” period, the majority of samples are distributed to members linked to UBiLim, which also temporarily affects the use rate. However, it is to be expected that this rate will increase in the near future, as many projects for which samples are stored are still in either collection phase or have not started analyses yet. Additionally, most of the collections in the UBiLim inventory are project-based and less at risk of “biohoarding” (38). Nonetheless, UBiLim's support has resulted in 29 publications based on samples stored in its facilities, a key performance indicator demonstrating operational sustainability of the biobank and demonstrating UBiLim's added value for translational research (39).

### The Future of UBiLim: and the Beat Goes on…

In November 2018, the Belgian biobank law eventually came into effect imposing that any human body material used for scientific research has to be obtained from a notified biobank (40). This evolution has increased the need for the UBiLim infrastructure, extending to other research ongoing in the three founding institutes and external parties in the Limburg area. As a result, many researchers that currently control their own active and historic sample collections request to be incorporated into the UBiLim biobank. Compared to the first quarter of 2018, an increase of 60% of collections/studies was observed in the first quarter of 2019. Although the legislation comes with its own set of challenges, it appears to induce the transition of researchers no longer setting up individually managed collections, but starting them within the context of already established biobanks. While this is a positive trend with respect to sample quality and harmonization, its impact on accelerating translational research will only become clearer in time.

With the public funding for the LCRP ending, Hasselt University, the Hospital East-Limburg, and the Jessa Hospital have each dedicated funding to transform it into the Limburg Clinical Research Center, in order to sustain the ongoing projects, among which UBiLim. The funding available to the biobank, however, would only cover 56% of the expenditure compared to the total costs of the biobank in 2018. Additionally, given the increased number of collections expected due to the changed legislation, the total cost in 2019 will be higher than that of 2018, potentially resulting in a higher deficit. Up to now, UBiLim has never charged fees for the services and/or samples delivered, mainly because of common funding sources, but also because of the limited budget available to researchers. However, it is clear that the sustainability of UBiLim needs to be addressed. The business aspect of biobanking, including sustainability, had recently received a lot of attention in the biobank community (41–44). However, while cost recovery appears to be an obvious solution, it has been reported to not contribute significantly to sustainability on its own (45). This can be partially overcome by providing a catalog of samples and improved “marketing” of the biobank (46, 47), but public funding is reported to be a critical component of an overall business plan (12, 48). UBiLim is currently investigating cost recovery as a model to at least partially cover operational costs, based on calculation tools available elsewhere (49, 50). Additionally, it is actively pursuing visibility of its infrastructure and its resources by displaying its catalog metadata on its website and in the publicly available BBMRI-ERIC directory (51).

## Conclusion

UBiLim, as it stands today, is a federated biobank, with processing and storage facilities at each of the three sites. Common procedures, corresponding to the medical laboratory quality standard and biobanking guidelines, are used to harmonize the activities and ensure comparable, qualitative samples, independent of the originating site. Funding has been a major challenge in each step of its evolution and remains the biggest issue for long-term biobank sustainability. To a lesser extent, the Belgian legislation and the operational cost of information management system are also concerns for smooth biobank operations. Nonetheless, the need for UBiLim's infrastructure is still apparent and increased growth is to be expected. Efforts are ongoing to improve the utilization rate as well as the sustainability of the biobank to ensure its long-term development. Several publications have arisen from the use of the samples, which may result in improved care and/or therapy of the patients involved. Nonetheless, through provision of professional biobank services, UBiLim serves as a facilitator for translational research in the Limburg area of Belgium that, given the fields of research, may have an impact on international patient care.

## Materials and Methods

Data on sample numbers, types, and diagnoses were acquired by running dedicated queries in the Biobank information management system Labvantage. Utilization rate was calculated as the percentage of the number of samples released annually vs. the cumulated number of samples in storage that year. The publications counted were those that effectively used samples that were processed and/or stored by UBiLim. Publications without biomedical content were excluded. Financial data were acquired from the accounting departments and divided into six main categories (personnel, operations, equipment, IT infrastructure, promotion/marketing, and overhead). Data were analyzed and visualized using Microsoft Excel 2016.

## Data Availability Statement

All datasets generated for this study are included in the manuscript/supplementary files.

## Author Contributions

LL, KV, EV, KU, SD, JP, VS, PS, and J-LR conceived of the presented idea and critically reviewed the manuscript. LL and KV designed the project, performed the data analysis, and co-wrote the paper.

### Conflict of Interest

The authors declare that the research was conducted in the absence of any commercial or financial relationships that could be construed as a potential conflict of interest.
